# Strengthening of oncology nursing education and training in Africa in the year of the nurse and midwife: addressing the challenges to improve cancer control in Africa

**DOI:** 10.3332/ecancer.2021.1209

**Published:** 2021-03-23

**Authors:** Naomi Oyoe Ohene Oti, Martjie de Villiers, Prisca Adejumo, Roselyne Okumu, Biemba Maliti, Nagwa Elkateb, Nazik Hammad

**Affiliations:** 1 National Radiotherapy, Oncology and Nuclear Medicine Centre, Guggisberg Avenue, Harley Street, PO Box KB369, Korle Bu–Accra, Ghana; 2 Accra Cancer Registry, Guggisberg Avenue, Harley Street, PO Box KB369, Korle Bu–Accra, Ghana; 3 African Organisation for Research and Training in Cancer, 1ST Floor, Birkdale 1 River Park, Gloucester Road, Mowbray, Cape Town, 7901, South Africa; 4 Adelaide Tambo School of Nursing Science, Building 5, Room 327, Staatsartillerie Road, Pretoria West, Private Bag x 680, South Africa; 5 Tshwane University of Technology, Building 5, Room 327, Staatsartillerie Road, Pretoria West, Private Bag x 680, South Africa; 6 University of Ibadan, Oduduwa Road, Ibadan, 900001, Nigeria; 7 Kenyatta National Hospital, PO Box 20723-00202, Nairobi, Kenya; 8 Cancer Disease Hospital PO Box 51337, Lusaka, 10101, Zambia; 9 Zambia Oncology Nurses Society, PO Box UTH73, Lusaka, 10101, Zambia; 10 National Cancer Institute, Kasr Eleini St. fom Elkhalig, Cairo, 1196, Egypt; 11 Queens University, 99 University Ave, Kingston, ON K7L 3N6, Canada; a https://orcid.org/0000-0002-1433-0364; b https://orcid.org/0000-0002-88109144; c https://orcid.org/0000-0003-3065-8308; d https://orcid.org/0000-0002-2459-8532; e https://orcid.org/0000-0002-2552-7031; f https://orcid.org/0000-0002-3961-1736; g https://orcid.org/0000-0003-3963-5815

**Keywords:** oncology nursing, education, training, cancer care, Africa

## Abstract

The Cancer burden in Africa is increasing. Nurses play a pivotal role in health care systems and find themselves in a key position to engage with patients, communities and other health professionals to address disparities in cancer care and work towards achieving cancer control in Africa. The rapidly evolving nature of cancer care requires a highly skilled and specialised oncology nurse to either provide clinical care and/or conduct research to improve evidence-based practice. Although Africa has been slow to respond to the need for trained oncology nurses, much has been done over the past few years. This article aims to provide an update of Oncology nursing education and training in Africa with specific focus on South Africa, Ghana, Nigeria, Kenya, Zambia and Egypt. Mapping oncology nursing education and training in Africa in 2020, the International Year of the Nurse and the Midwife, provides an opportunity to leverage on the essential roles of the oncology nurse and commit to an agenda that will drive and sustain progress to 2030 and beyond.

## Background

Traditionally, cancer was not Africa’s most concerning health care problem [1]. However, over recent years, with an increasingly ageing population and more westernised lifestyle changes, cancer has become a public health threat [2]. In 2018, the African continent has seen an estimated 752,000 people newly diagnosed with cancer; that is 4% of the total global cancer incidence rate; with cancer deaths totaling 506,000 for the same period [3, 4]. Moreover, it is projected that the cancer burden will increase by more than 60% by 2030 in low-and middle-income countries (LMIC) [2]. Of the people diagnosed with cancer in sub-Saharan Africa, approximately 80% present with advanced disease at the time of diagnosis [1]. LMIC’s ability to respond to the cancer burden is hampered by the limited resources, especially specialised healthcare personnel [1, 5, 6]. In 2015, it was found that there were only 102 cancer treatment centres on the continent, of which 38 were found in South Africa [7].

There is pressing demand for specialised Oncology personnel, especially nurses. Nurses, who are seen as the backbone of the healthcare system [8], are often the first point of entry to the system and play a pivotal role in cancer control programmes [9]. An effective, skilled oncology nursing workforce can provide care throughout the cancer continuum – from prevention, early detection, various treatment modalities to survivorship care and ultimately end-of-life care [10] thus contributing to both a reduction of cancer burden and improvement in patients’ outcomes [6]. Research has shown that having a highly competent and skilled nurse who provides quality care leads to reducing patient mortality [5, 11]. Despite the key contribution nurses can offer to control cancer, LMICs devote few resources and pay limited attention towards the development of the cancer nursing workforce [10]. Out of 22 countries, six reported having no oncology trained nurses [9].

African countries such as South Africa and Egypt have been providing specialised oncology education and training for many years; however, in recent years, several other countries have since developed formal education and training programmes leading to certification in oncology nursing [12].

## Significance of specialisation in oncology nursing

Although nurses are key providers of cancer care across the spectrum, the effectiveness of care is dependent on the nurse’s acquired knowledge and skills [13, 14]. In addition, nurses are capable of designing and delivering innovative culturally acceptable cost-effective cancer control packages by identifying and closing the gaps on missed opportunities, thus providing for the holistic approach in caring for these patients [6, 15]. Therefore, the need for effective training of the African nurses to provide expert oncology nursing care for the continent is paramount by developing and implementing innovative interventions and strategies to enhance oncology nursing education and training in LMICs.

## Purpose

This article aims to provide an update on oncology nursing education and training in Africa, challenges and the way forward with specific focus on South Africa, Ghana, Kenya, Zambia, Nigeria and Egypt. These countries have formal education and training programmes accredited by their respective regulatory bodies and are representative of the selected regions in Africa.

## Method

This article was written by representatives from the different countries involved, who could provide accurate information from the educational institutions providing oncology nursing education and training as well as the regulatory bodies and challenges experienced. Furthermore, a literature search was conducted using Public/Publisher MEDLINE, Scopus, Web of Science and Cumulative Index to Nursing and Allied Health Literature as databases and ‘education and training’, ‘Oncology OR Cancer nursing’ and ‘Africa’ as search words. The authors also requested the members of African Organisation for Research and Training in Cancer (AORTIC) Nursing group and the African Cancer Nurses Network for input with regard to Oncology nursing education and training programmes for information pertaining to education and training available in their countries. Data were obtained electronically via use of Telegram (a social media app) to reach and obtain information from the African Cancer Nurse Network (171 nurses representing 10 countries) and a direct call to members of the AORTIC Nursing group. Participants were asked whether or not an oncology nursing programme was currently being offered in their countries and details of the programme; in terms of duration, type of certificate or degree offered, whether it is postgraduate and whether or not the programme/s were accredited by the appropriate regulatory body. This was supported by a telephonic interview of key personnel in the various countries represented on the AORTIC Nursing group. In the instance where a specific country was not represented on the group, the country was contacted vial telephone through its representative in the main AORTIC network as to obtain information by means of direct contact. A survey was posted on the Telegram social media group. The information obtained from both the survey and the direct phone contact were combined and captured on an Excel spreadsheet and plotted on a map of Africa. This paper highlights the evolution of Oncology Nursing in Africa, the role of oncology nursing, the selected country-specific experience, mode of education, available oncology nursing education and training, challenges and enabling actions.

## Results

### Role of the oncology nurse

The European Oncology Nursing Society defined a cancer nurse as a registered nurse who has the mandate and full responsibility to provide crucial nursing care to people affected by cancer based on his/her evidence-based, specialised ethical and personal knowledge and skills [16]. These nurses have unique roles and responsibilities which continuously change as the field of oncology continues to evolve [17]. The roles are diverse and range from that of creating cancer awareness and cancer advocacy, which includes cancer prevention activities, coordination of care, health education, symptom management and supportive care [18, 19]. In addition, effective nursing administration, training and conducting research to improve evidence-based care are some of the broad roles the oncology nurse fulfils. These roles are enhanced by the Advance Practice Nurses who are professional nurses with additional postgraduate training (minimum of a Master’s degree) with expert knowledge base, complex decision making skills and clinical competences in the field of oncology nursing licenced by appropriate regulatory body to practise as such [16, 20]. The Oncology nurse can practise in different health care settings: ambulatory care, acute care and community services [21].

The scope of competencies includes amongst others, advanced knowledge and skill in a variety of treatment modalities such as surgery, systemic therapy (chemotherapy, hormonal and targeted therapy) and radiotherapy, the assessment and management of acute and chronic symptoms of cancer, the treatment side effects, guidance of the patient and family through the cancer continuum, advocating for and participating in policy formulation and conducting research [20]. In addition, effective leadership is of utmost importance in order to influence patient outcomes by increasing the quality of care and advocating for cost-effective interventions for patients/clients [17, 22, 23].

## Evolution of oncology nursing education and training in Africa

Similar to the high income countries (HIC), around the mid-20th century, surgery and radiotherapy were the main treatment modality for cancers in Africa until the 1970s when additional therapy such as chemotherapy was added and was solely administered by physicians [24–27]. Patients presented with late stage cancers and the role of the nurse was limited to inpatient care of hospitalised surgical patients and terminally ill patients. In the late 70s and 80s, most nurses in Africa joined in the administration of chemotherapy through on-the-job training [27]. This increased nursing contribution to cancer care. Some of the nurses in Africa had training in the UK and North America and upon return took up the mantle of ‘Train-the-Trainer’ initiative to train others nurses in the administration of chemotherapy to patients, managing side effects of both radiation and chemotherapy and health education of patients and their families about disease, treatment process and side-effects. Anecdotal data suggest that in the 1980s, nurses in countries such as Egypt and South Africa held their first national meetings as nurses in cancer care, respectively. This propelled the start of formal oncology nursing education and training in both countries. However, during the 90s, most education and training in Africa were done through the provision of in-service training [15]. While essential, such training did not provide nurses with the skills to reach their full potential in maximising patient outcome. Over the years, some HICs afforded opportunities in education and training with the help of several non-governmental organisations in an attempt to strengthen oncology nursing practice in Africa [28, 29]. These education and training sessions were often provided *ad-hoc* by experts in the field and were seldom followed-up to ensure sustainability of the acquired knowledge and skill [15]. Recently more African countries have initiated the development of oncology nursing curricula which are offered at different levels [30, 31].

### Country-specific situation analysis

#### South Africa

Oncology nurse education and training started in the 90s during which time there were an increase in oncology care facilities and a need arose to recognise the specialised skill needed to practise in such an environment. Since then, formal training programmes in oncology nursing are available at different levels at various higher education institutions; two regular universities, two universities of technology and one nursing college. The qualifications are offered at Honor’s-, Master’s-, Bachelors and Diploma level, respectively. Unique to these programmes are a comprehensive module on principles of palliative care nursing. Up to the end of 2018, a total of 641 Oncology nurses were on the South African Nursing Council (SANC) register [32]. Programmes are accredited by the SANC, as well as the Council for Higher Education.

Currently these programmes are in the process of being phased out due to nursing education and training being moved from the Department of Health to the Department of Higher Education and Training and the need to align nursing degrees to the National Qualification Framework (South Africa, 2013). The new qualification to be rolled out within the next year or two will be known as a Postgraduate Diploma in Oncology and Palliative Care Nursing. The holder of a Postgraduate Diploma will be known as a nurse specialist in Oncology and Palliative care nursing. The training allows vertical professional progress to a Master’s degree in nursing (Advanced specialist nurse).

In preparation for the new programme, academics and representatives from Oncology nursing practice came together and developed a scope of practice which included the competencies and minimum skill set of an Oncology and Palliative care nurse in South Africa [32]. The exit level outcomes of the new Postgraduate Diploma in Oncology and Palliative care nursing were based on these newly defined competencies. Considering the Cancer burden in South Africa and the number of trained oncology nurses needed, efforts must continue to up-scale training. Funding for this programme is basically self-funded but some students apply for student loans. These training programmes are also a hub for international students all over sub-Saharan Africa, who are mostly sponsored by International Atomic Energy Agency (IAEA) or their Ministries of Health.

#### Ghana

Ghana had its first oncology centre in 1997 and the second in 2005, established through a collaboration between the Government of Ghana, IAEA and the Ghana Atomic Energy Commission [33]. With this collaborative effort, two nurses were trained abroad; one for each centre and upon return, embarked on ‘Train the Trainer’ initiative to train other registered nurses on the job to help give specialised care to people with cancer and their families. Others subsequently followed to attend oncology nursing education and training abroad or by means of online educational programmes hosted by academic institutions in Europe or North America. These programmes provided the nurse with either a certificate, diploma or degree in oncology nursing. In-service training was also done. The limited access to training in this specialised area and inadequate trained health professionals in oncology was increasingly becoming a major challenge. The skills gap created made it crucial for oncology nursing specialisation, a necessity for effective patient care and quality outcome. In 2011, the Parliament of Ghana enacted the Specialist Health training and Plant Medicine Research Act, 2011 (ACT 833) [34, 35]. Part three of its provisions paved way for the birth of Ghana College of Nurses and Midwives (GCNM), whose mandate is to provide postgraduate specialist education and training in Nursing and Midwifery [35]. The College started with eight courses, of which Oncology nursing was one of them [36]. The training is credentialed by the Nursing and Midwifery Council (NMC) of Ghana and the eligibility for this programme is that, a candidate should have at least a Bachelor of Science in Nursing with about 3 years practising experience [37]. Also having a master’s or a PhD in nursing or related area is an advantage [37]. Training started in 2015 and the first three cohorts in oncology nursing delivered a total of 12 graduates (2018, 2019 and 2020, respectively) who were awarded a postgraduate certificate of membership from GCNM and credentialed to practise as Nurse Specialists [37]. Also, these cadre of nurses are being prepared to be clinicians and academicians [35]. Furthermore, to allow career advancement, graduates are allowed fellowship and awarded a fellowship certificate to progress from Nurse Specialist to Senior Specialist then to Nurse Consultant [37]. Currently, the total number of local formal trained oncology nurses is 45 (12 Nurse Specialists and 33 nurses in training). This numbers are inadequate in the face of the growing Cancer incidence in Ghana [3, 38].

The main challenge is limited oncology nursing faculty to support training. This programme is self-funded and sometimes as scholarships by pharmaceutical companies such as Hoffmann La Roche [39].

#### Kenya

The first oncology nursing education and training in Kenya started at the University of Nairobi (UoN) in the year 2010 with Master of Science in Oncology Nursing (MScN) degree and has been ongoing for a period of 10 years. Prior to this a few oncology nurses were trained outside the country but the majority learnt through on the-job training. In 2016, the higher national diploma training equivalent to post basic oncology nursing training was commenced and was accredited by the nursing council of Kenya.

The launch of the Kenya National Cancer Control Strategy (NCCS, 2017–2022,) reinforced the need to improve the human resources for cancer care [40]. Many institutions have leveraged on the NCCS to develop curriculums and initiate training. Training is provided at post-basic and master’s level. Post-basic graduates are known as oncology nurse generalists, while Master level graduates are Oncology Nurse Specialists. A total of 77 nurses have successfully completed the post-basic programme, whereas 11 graduated as MScN oncology nurse specialists. At present, 46 and 23 nurses, respectively, are in training at different colleges and universities across the country.

Kenya Oncology Nurses Chapter also provides a learning platform by organising annual scientific conferences which are attended by nurses around the country. Short courses on chemotherapy safety (Chemo-safe) training by various partners have gone a long way in capacity building of the multidisciplinary team especially the nurses working at the chemotherapy units [41]. At present the greatest need is to develop a clear job description of specialised oncology nurses to assist with career pathway progression. There is need to strengthen and develop the pool of specialised oncology nurses to enable them to empower, educate and train prospective oncology nurses and to conduct research that would influence policies and practice around oncology nursing. The trained oncology nurses are few, yet the training is mainly funded by out of pocket and sometimes funded by student loans from higher education loans board depending on the level of training. In few occasions, students may be funded by partners like Pharma Companies.

#### Zambia

Zambia has one dedicated oncology hospital, the Cancer Diseases Hospital, which opened in 2006, during which time there were no locally available specialised oncology nurses. The hospital offers chemotherapy, radiotherapy, surgical oncology, pediatric oncology, nuclear medicine and palliative care services. In 2007, IAEA sent nurse experts to help train the first registered nurses at the hospital. Thereafter, the head of nursing completed the Oncology Nursing Society’s (ONS) Chemotherapy and Biotherapy programme, with several other registered nurses following suit to complete the online ONS Radiation Oncology Nursing certificate. Registered nurses were also sponsored to attend education and training in South Africa.

In 2009, the hospital’s Nursing department wrote a concept note on developing a local oncology nursing training and lobbied the Ministry of Health (MOH) and General Nursing Council of Zambia (GNCZ) for support. The oncology nurses also came together and registered the Zambia Oncology Nurses Society in 2016, which was instrumental in driving the agenda for a local oncology nurse education and training programme development. Several local and international stakeholders were involved in the development of the curriculum spearheaded by the GNCZ. In 2017, the first curriculum for a 2-year Bachelor of Science in Oncology Nursing was completed and introduced at the university, it enrolled nurses who had at a minimum the Registered Nurse Diploma and at least 2 years’ of clinical experience. Recognition of prior learning is also considered which allows trainees to move from college nursing programmes via foundation nursing straight to post-basic oncology programme without prior years of practice experience. In 2018, the second curriculum for a 1-year Advanced Diploma in Oncology Nursing was completed and the first students enrolled in 2020 at the Levy Mwanawasa Medical University.

A total of 19 registered nurses have become specialised Oncology Nurses between 2008 and 2019, having trained in South Africa which was sponsored by the government of Zambia. To date, three nurses have obtained Masters in Oncology and Palliative Nursing from South Africa.

#### Nigeria

Until recently, there were no formal accredited oncology nursing education and training programmes in Nigeria. Nurses working in oncology units in various settings rely on in-service training, short-term workshops, conference attendance and learning on the job [6]. The few nurses, who were specialised oncology nurses had done so abroad [6]. Realising the need to meet the burden in cancer care, the Nigerian Federal MOH established a National Cancer Control Plan (NCCP) for a 5-year period between 2018 and 2022 as a commitment to safeguarding the health of the citizens [42]. An educated oncology nursing workforce is paramount to the successful implementation of this plan.

A post-basic Diploma in Oncology nursing programme, which was recently accredited, is the only available oncology nursing education programme in the country and is hospital-based. This programme is offered over a period of 1 year with candidates for admission being nurses registered with the NMC of Nigeria.

The graduates of this programme are known as oncology nurses and practise in oncology units but without any special remuneration or benefits accruing to them as a result of the training. Before accreditation of the programme, a total of 136 nurses have been trained. Currently a total of 24 nurses are enrolled in the accredited oncology nursing training programme with the admission of a new set being halted by the lockdown.

There is an ongoing plan by the NMC of Nigeria to transform oncology nursing from its present status of hospital-based post basic programme to postgraduate programme within the universities. At the University level, it is currently offered as a course within medical surgical sub-speciality. However, this is yet to be finalised as at the time of this write up.

Presently, there is not yet a clearly defined career pathway specifically for Oncology nurses in spite of diverse opportunities for professional growth and development.

Considering the cancer burden in Nigeria, the number of trained oncology nurses is inadequate. Funding is primarily by out of pocket and sometimes by employers of the trainees as scholarships.

#### Egypt

In 1996, the Technical Institute of Nursing, affiliated to the National Cancer Institute (NCI), Cairo University, was established to develop a training programme, preparing graduates to provide total care for cancer patients in Egypt. The 2-year programme, followed by a 6 months’ internship period, prepares nurses in areas of general Oncology nursing as well as a variety of sub-speciality areas, for example, palliative care or stoma care. Graduates earn a Diploma in Technical Nursing (DTN) certificate, an associate degree in cancer nursing from Cairo University and are committed to practise as a staff nurse at the NCI, Cairo. Recent introduction of new Bylaws and Regulations released by the Supreme Council of Egyptian Universities stipulated that all nursing Education Institutes should follow the same bylaws. These requirements necessitated changes in the current programme, implying that enrolled students complete a 2-year programme in General nursing followed by a 6-month internship in cancer nursing to earn a DTN. This will be followed by a further 2-year specialisation in Oncology nursing to obtain a Bachelor degree in Oncology nursing from the Supreme Council of Egyptian Universities and practise as an Oncology nurse specialist or Oncology nurse educator. This new programme should commence in the 2020/2021 academic year. Other prospective changes also include a Master’s degree in Oncology nursing and to expand the programme to other education institutions. So far about 500 nurses with DTN associate degree, 35 with Bachelor Oncology Nursing, 5 with Master degree in Oncology Nursing and 4 with a Doctor of Science as faculty. Continuity in the provision of quality cancer care is ensured through continuous education and training workshops and other training programmes, offered to nurses practising at the NCI, Cairo and other cancer centres and cancer units in general hospitals. Sometimes, workshops are arranged in collaboration with Union of International Cancer Control and Middle East Cancer Consortium (MECC). Also, there is an annual Oncology nursing educational conference presented in Cairo for all nurses working in cancer centres and units in Egypt. Partnering with associations abroad has led some nurses to attend training in cancer centres in the USA, UK and Jordan as well as oncology nursing and palliative care programmes and workshops with the MECC in different Middle East countries as Turkey, Cyprus and Oman. At NCI, Egypt Oncology Nursing training is funded through Scientific Activities fund. The only barrier is lack of funding to send nurses abroad for training or to recruit international pioneers to present training locally.

## Mode of education, admission requirements and programme duration

Specialisation in oncology nursing requires a nurse to receive education and training to acquire advanced knowledge and skill up to the PhD level in the principles and practice of oncology nursing, combined with internship for a certain period of time [19]. Thereafter, the nurse obtains certification from and is licensed to practise by the professional regulatory body from the specific country.

Formal education and training of oncology nursing in Africa is mostly done at the university or college level and the practicums are conducted in designated teaching hospitals which have cancer centres. The level of training varies from country to country and includes a post basic diploma/degree offered as a 1-year full time or 2 years’ part time programme, Masters of Science in oncology nursing is offered over a period of 2 years or a 3-year postgraduate residency programme. For a person to be eligible to enrol in formal oncology nursing education and training at various levels, one needs to be a qualified nurse with varying years of practising experience.

The principle of andragogy is applied to enable learners to meet the educational requirements and thus all programmes offer didactic learning as well as a clinical practicum component. The clinical learning experience is further enhanced by clinical and academic presentations from hospital staff which is found to be beneficial to both clinical staff and students. Clinical facilitation is done by a multidisciplinary team which includes oncology nurse educators, oncologists, medical physicists and other oncology health professionals at the hospitals and from the universities. All the training programmes include delivery of research training appropriate for the level of training. Training also includes modules on palliative care nursing principles.

## Available oncology nursing education and training in Africa

There had been some strides in some countries in Africa developing their own curricula to train oncology nurses at various levels. From the survey and direct contact, we found out that 16 countries had some form of oncology nursing education and training going on at different levels; Five in East Africa, three in North Africa, two in West Africa and two in Southern Africa. The programmes in the other four countries are in their developmental stages. Central Africa currently has no ongoing formal training.

**Figure 1.**
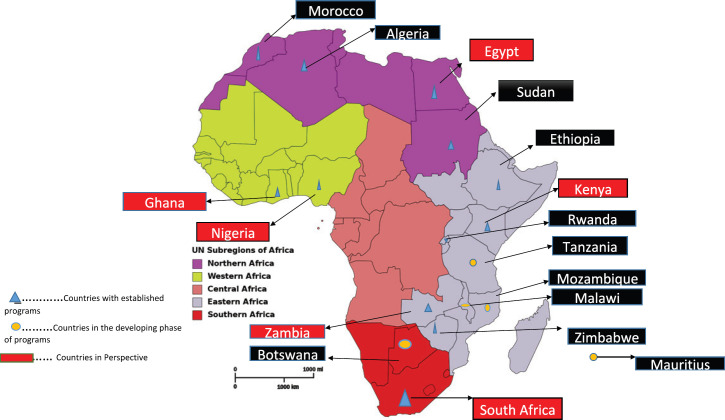
African map showing countries with oncology nursing education and training.

Table 1 shows the various African countries with oncology nursing education and training programmes, the various levels at which these programmes are offered and the development stages of the curricula. Figure 1 shows a map of Africa indicating the various countries with ongoing oncology nursing programmes.

## Challenges to oncology nursing education and training in Africa

### Policies, leadership and funding

Immense success has come from nurse leadership in Africa when looking at palliative care programmes in Uganda and Rwanda [9]. The AORTIC Nursing Special Interest Group is currently developing standard competencies for oncology nursing. Hopefully, more success is yet to come with the implementation of the National Comprehensive Cancer Network Harmonised Guidelines^TM^ for sub-Saharan Africa [43]. However, oncology nursing speciality education and training has not yet been implemented by all LMICs as proposed by the Global Action Plan for the Prevention and Control of Non-Communicable Disease: 2013–2020 [44]. Also, there are no standardised scope of practice, nor clearly defined competencies which can inform education and training and ultimately practice [5]. In most countries, nurses do not form part of the required leadership positions in government to be able to influence cancer care policies to include a nursing perspective [10]. Nurses are not able to articulate specific professional matters, drive patient care as they desire hence they end up following decisions made by others. This is not only a problem at government level, but also at institutional level. Cancer centres rarely recognise nurses in leadership and planning [10, 23]. Historically, nursing has been seen as a low status profession with poor wages, limited in professional enhancement and exposure to global learning [45, 46].

Unfortunately, policy links directly with funding opportunities. Financial support for oncology nursing training programmes is scarce and is influenced by the social, economic setting and political will of the country [47, 48]. The prevalence of self-funding of training is unfortunate. Some funding institutions are not interested in funding nursing education, with the feeling that nurses are not the key factor in deciding on patients’ treatment outlines and outcomes of patients [49] and is evident by the dominance of the medical profession and the creation of professional silos [50].

### Educational pathways

Oncology nursing education and training is not yet well recognised in Africa and is a major obstacle to broaden and upskill the oncology nursing workforce [9]. In general, nurses in LMICs have insufficient knowledge of cancer care and the situation is exacerbated by the fact that there is limited opportunity for specialised education. Informal training programmes are available [12], but insufficient and unsustainable [9]. There is also lack of qualified faculty to teach oncology nursing programmes and few doctoral programmes in oncology nursing to prepare faculty [6]. With the constantly changing oncology environment, continuing education programmes are of utmost importance, not only for development and skills, but also to ensure job satisfaction. However, with the low number of specialised oncology nurses’ available, development of continuous development programmes is also difficult [5].

### Research

**Figure 2.**
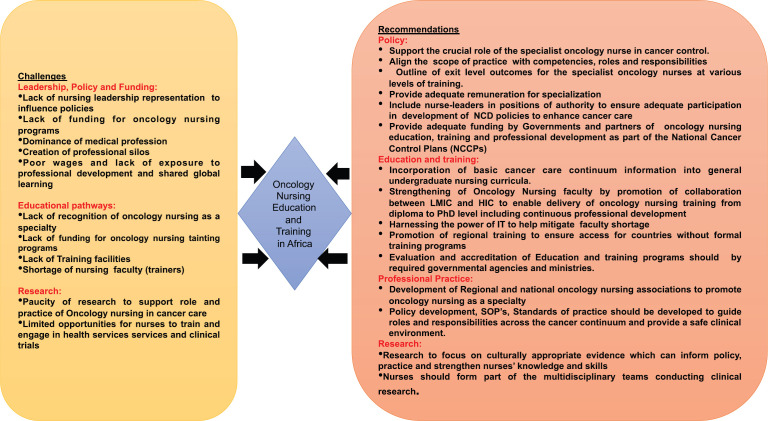
A summary of the challenges and way forward of oncology nursing and education in Africa.

Funding is needed for research, but the reverse is also true, research is also needed to drive funding. There is paucity of research output to support the key role nurses play in cancer control programmes [51, 52] . Research informs education and training as well as evidence-based practice. The International Council of Nurses conference in 2019 reiterated the importance of having reliable data and evidence to ensure a reliable, healthy workforce to and inform governments how and where to invest in terms of health and health outcomes [53]. A summary of the challenges and recommendations are shown in Figure 2.

## Recommendations

### Policy

The role of the specialist oncology nurse in cancer control should be recognised and supported by a thorough review of the legal scope of practice of the nurse. The scope of practice should be aligned with competencies, roles and responsibilities and exit level outcomes for the specialist oncology nurses at various levels – certificate, diploma, bachelors, masters and Doctorate [10]. Also, nurse-leaders should lobby for their inclusion in positions of authority at institutional as well as governmental level to advocate for the valuable contribution nurses can make in the cancer control efforts [5]. Governments, non-governmental organisations, pharmaceutical companies and higher education institutions need to formulate plans to secure funding for oncology nursing education and training as part of their NCCPs [10]. Although not proven to ensure attrition of specialised nurses, provision should be made to adequately compensate and remunerate specialised oncology nurses [5]. AORTIC has a specially designated seat for oncology nursing in its executive council and is promoting the scaling up of oncology nursing in Africa.

### Education and training

Learning about the cancer continuum and the role a nurse plays in cancer control should already be incorporated into the general undergraduate nursing curriculum; this will assist with bridging the gap in cancer knowledge [10]. Nursing faculty should be equipped with knowledge and skill up the PhD level to develop oncology nursing curriculum so as to be able to train oncology nurses and collaborate with HIC to strengthen further specialisation and training opportunities [5, 10]. There is also the need to harness the power of Information Technology such as use of online-platforms, to help compensate for the shortage in nursing faculty in Africa [54–56]. Regional training programmes should be promoted so that countries who do not have formal education and training in oncology nursing can access training to deliver cancer care to their populace.

Education and training programmes should be evaluated and accredited by required governmental agencies to ensure good standard but also to obtain by-in from the required ministries [5].

### Professional practice

Regional and national oncology nursing associations should be developed and nurses committed to oncology nursing should join. Nurses in cancer care should be sitting at the table especially in organisations where all health professionals in cancer care meet to engage in activities to promote cancer control. The focus of these associations should be to raise awareness in terms of the need for oncology nursing specialisation and creating research opportunities [10]. At present, few countries in Africa have national oncology nursing associations [57–59]. Still in their developmental phase, there have been various social media groups, providing oncology nurses from all across Africa, with helpful oncology nursing tips, sharing best-practice and providing support.

Policy development, standard operating procedures and standards of practice should be developed to guide roles and responsibilities across the cancer continuum and provide a safe clinical environment [5, 10]. At present, a process of developing oncology nursing competencies for Africa has been set in motion by organisations such as the International Society of Nurses in Cancer Care and AORTIC Nursing group [10].

### Research

Oncology nursing research output should be prioritised to provide evidence that is culturally appropriate and can inform evidence based practice, policy and strengthen nursing knowledge [10, 17, 60, 61]. Thus nurses should be integral members of multidisciplinary teams conducting health services research or clinical trials in order to expand knowledge about best practices and clinical application of research findings [5, 10].

## Conclusion

Nursing is the largest group in the health care sector, accounting for approximately 59% of healthcare professionals. In a highly connected, team-based and digital era, no global health agenda can be realised without concerted and sustained efforts to maximise the contributions of the oncology nursing workforce in line with the State of the world’s nursing report, 2020 [62]. Mapping oncology nursing education and training in Africa in 2020 as the International Year of the Nurse and the Midwife provides an opportunity to leverage on the essential roles of the oncology nurse and commit to an agenda that will drive and sustain progress to 2030 and beyond [63, 64].

Investing in the massive upscale of nursing education and training is urgently needed. Enabling actions are faculty development, infrastructure and curricular upgrades, mitigating identified challenges in the pathways of education and training, research, policy and professional practice. Addressing oncology nursing needs and strengthening oncology nursing leadership will contribute to the effectiveness of cancer care and improve outcomes in Africa.

## List of abbreviations

LMIC, Low-middle income countries; AORTIC, African Organisation for Research and Training in Cancer Care; ONS, Oncology Nursing Society; HIC, High income countries; SANC, South Africa Nursing Council; IAEA, International Atomic Energy Agency; GCNM, Ghana College of Nurses and Midwives; NMC, Nursing and Midwifery Council; MSc.

N, Masters of Science in Nursing; GNC, General Nursing Council; NCI, National Cancer Institute; MOH, Ministry of Health; DTN, Diploma in Technical Nursing; MECC, Middle East Cancer Consortium; PhD, Doctor of Philosophy; UON, University of Nairobi

## Authors’ contributions

All authors contributed to the final version of the paper.

## Conflicts of interest

The authors declare that there are no conflicts of interest.

## Funding statement

This research received no specific grant from any funding agency in the public, commercial or not-for-profit sectors.

## Figures and Tables

**Table 1.** Countries with Oncology Nursing training and education in Africa.

	Country	Level of training/education				
		Diploma (Higher National Diploma)	Bachelor’s	Post Registration Bachelor’s degree^a^	Postgraduate Membership Certificate^b^	Master’s degree
1	Ghana				✓	
2	Nigeria	✓				
3	South Africa			✓		✓
4	Zambia	✓	✓			
5	Zimbabwe		✓			
6	Kenya	✓				✓
7	Egypt	✓	✓			✓
8	Ethiopia		✓			✓
9	Sudan		✓			
10	Rwanda		✓			✓
11	Morocco		✓			
12	Algeria		✓			
Countries with developing programs and level of training						
1	Botswana					✓
2	Malawi			✓		
3	Tanzania					✓
4	Mauritius					

a Post Registration Bachelor’s degree also known as Postgraduate Diploma

b Postgraduate Membership Certificate (3-year Postgraduate Oncology Nursing Residency)
